# Genome-Wide Identification and Analysis of *ZF-HD* Gene Family in Moso Bamboo (*Phyllostachys edulis*)

**DOI:** 10.3390/plants12234064

**Published:** 2023-12-03

**Authors:** Feiyi Huang, Jiaxin Wang, Chao Tang

**Affiliations:** 1 Co-Innovation Center for Sustainable Forestry in Southern China, Bamboo Research Institute, College of Life Sciences, Nanjing Forestry University, Nanjing 210037, China; 2 State Key Laboratory of Crop Genetics & Germplasm Enhancement and Utilization, Centre of Pear Engineering Technology Research, Nanjing Agricultural University, Nanjing 210095, China

**Keywords:** moso bamboo, genome-wide, ZF-HD, abiotic stress, expression pattern

## Abstract

Zinc finger-homeodomain (ZF-HD) proteins play essential roles in plant growth, development and stress responses. However, knowledge of the expression and evolutionary history of *ZF-HD* genes in moso bamboo remains limited. In this study, a total of 24 *ZF-HD* genes were found unevenly distributed on 12 chromosomes in moso bamboo (*Phyllostachys edulis*). Phylogenetic analysis indicated that *PeZF-HD*s were divided into two subfamilies: ZHD and MIF. The ZHD subfamily genes were further classified into seven groups according to their orthologous relationships among the rice and *Arabidopsis ZF-HD* gene family. The gene structures and conserved motifs of *PeZF-HD*s were analyzed. Whole-genome duplication (WGD) or segmental duplication promoted the evolution and expansion of the moso bamboo *ZF-HD* gene family. Ka/Ks ratios suggested that the twenty-four duplication pairs had undergone purifying selection. Promoter analysis showed that most *PeZF-HD*s contained *cis*-elements associated with stress responses and hormones. Expression analysis demonstrated that many *PeZF-HD*s were responsive to abiotic stress treatment. Overall, this work investigated *PeZF-HD* genes in moso bamboo using bioinformatic approaches. The evolutionary research on gene structure, motif distribution and *cis*-regulatory elements indicated that *PeZF-HD*s play distinct roles in biological processes, which provides a theoretical basis for exploring the physiological functions of *ZF-HD*s and selecting candidate stress-related genes in moso bamboo.

## 1. Introduction

Plants often face various abiotic stresses during their life cycle, including extreme temperatures, drought and salinity, which seriously limit plant growth, development, quality and yield [1]. The transcription factor is a key kind of regulatory protein which can bind to the promoters of downstream genes and regulate their expression to adapt to multiple stresses [2]. As plant-specific transcription factors, zinc finger-homeodomain (ZF-HD) proteins have aroused much interest as they play vital regulatory roles in plant growth and development, as well as various stress responses [3].

The ZF-HD protein contains the conserved ZF-HD_dimer domain (PF04770) which consists of two highly conserved domains: the N-terminal C2H2-type zinc finger (ZF) and the C-terminal homeodomain (HD) [4]. The ZF domain can not only bind DNA but enhance the protein–DNA interactions controlled by the HD domain to either enhance or repress transcription [5]. As a DNA-binding domain, the HD has approximately 60 conserved amino acids, which fold into a three-helix structure and specifically connect to DNA [6]. HD-containing proteins are also involved in protein–protein interactions and other regulatory functions. Based on the size, location and structure of the HD domain and its association with other domains, HD-containing proteins can be divided into six distinct families: finger-like domain-associated HD (PHD finger), WUSCHEL-related homeobox (WOX), knotted-related homeobox (KNOX), zinc finger motif-associated HD (ZF-HD), leucine zipper-associated HD (HD-Zip) and bell-type HD [7]. The zinc finger structure contains a single zinc ion surrounded by two pairs of conserved cysteine and/or histidine residues [8]. According to the number, spacing pattern and nature of zinc-binding residues, zinc finger structures are classified into different types, such as C2H2 and C3H [9]. ZF-HD proteins can be divided into ZF-HD and mini zinc finger (MIF) groups based on phylogeny. MIF groups only contain the ZF domain without the HD domain, and are involved in integrating signals from GA, auxin, abscisic acid (ABA) and brassinosteroid [10]. However, the origins of and evolutionary relationship between these two groups remains unclear.

ZF-HD proteins were first discovered in the C4 plant *Flaveria* [5]. Subsequently, *ZF-HD* genes were reported in many plant species, such as *Arabidopsis*, rice, maize and tomato [11,12,13,14]. There has been increasing evidence suggesting that ZF-HD genes play important roles in responding to adverse stresses, except for plant growth and development [12]. Multiple *ZF-HD*s in *Arabidopsis* are involved in stress responses to adversity, and similar findings have also been reported in wheat and tomato [15,16,17]. *AtZF-HD1* can bind to the promoter of early response to dehydration stress 1 (ERD1) and its expression is inducible by drought, salinity and ABA. An overexpression of *ZF-HD1* and *NAC* enhances drought resistance in *Arabidopsis* [15]. A silencing of *SlZF-HD13* decreases the salt and drought tolerance of tomato [17]. In rice, four *ZF-HD*s can bind to a *DREB1B* promoter and activate its expression under drought and cold stress [12]. Most *BraZF-HD*s are induced by abiotic stress, indicating their important roles in controlling stress response [18]. Except for in the model plant, the identification and functions of *ZF-HD*s have also been broadly elucidated. There have been, respectively, 49, 50, 22, and 32 *ZF-HD* genes identified in four cotton genomes, in which gene duplication drove the expansion of the *ZF-HD* gene family during the divergence of the *Gossypium* species [19]. Twenty *FtZF-HD* genes have been identified in Tartary buckwheat (*Fagopyrum tataricum*), which have been further divided into five subfamilies [20]. In quinoa, 23 *CqZF-HD*s were detected in its genome, and *CqZF-HD14* was able to promote its resistance to drought by regulating the expressions of *CqNAC79* and *CqHIPP34* [21]. Although *ZF-HD* genes have been widely studied in many plant species, reports on *ZF-HD* genes in moso bamboo (*Phyllostachys edulis*) are limited.

Moso bamboo belongs to the Bambusoideae subfamily of the Poaceae family and is an important non-timber forest product. It is the most widespread bamboo species in China and has the highest economic, ecological and ornamental value [22]. Due to its propensity for fast growth, it may contribute to water and soil conservation and climate regulation [23]. Disadvantageous environmental and climatic conditions restrict the growth and development of moso bamboo, leading to severe yield losses. Recently, the genome of moso bamboo has been published, providing the foundation for a genome-wide analysis of *ZF-HD* genes in moso bamboo [24]. Herein, 24 putative *PeZF-HD* genes were identified and a comprehensive analysis was performed, including analyses of physicochemical properties, phylogenetic relationships, gene structure, conserved motifs, chromosomal localization, cis-elements, expression profiling and evolutionary analysis. These results will improve the understanding of the *PeZF-HD* gene family and provide the key candidate genes related to abiotic stress in moso bamboo.

## 2. Results

### 2.1. Identification of ZF-HD Genes in Moso Bamboo

*P. edulis ZF-HD* genes were screened by BLASTP and HMM. After excluding the redundant sequences, SMART was performed to confirm the presence of ZF-HD_dimer domains (Table S3). A total of 24 ZF-HD proteins were identified as *PeZF-HD* genes, and were named PeZF-HD1–PeZF-HD24. The characteristics of these *PeZF-HD*s are shown in Table 1, including the length of CDS and protein, molecular weight, theoretical isoelectric point, exon number, chromosome location and subcellular localization. The length of CDS of *PeZF-HD* genes varied from 252 bp to 1212 bp. The protein sequence lengths ranged from 83 to 403 amino acids, the molecular weights spanned 8.59 to 43.36 kDa, and the isoelectric points had a range of 5.90 to 10.30. The number of exons in *PeZF-HD* genes ranged from one to three. The predicted subcellular localization results indicated that all PeZF-HD proteins were located in the nucleus.

### 2.2. Phylogenetic Analysis of the Moso Bamboo ZF-HD Gene Family

**Figure 1:**
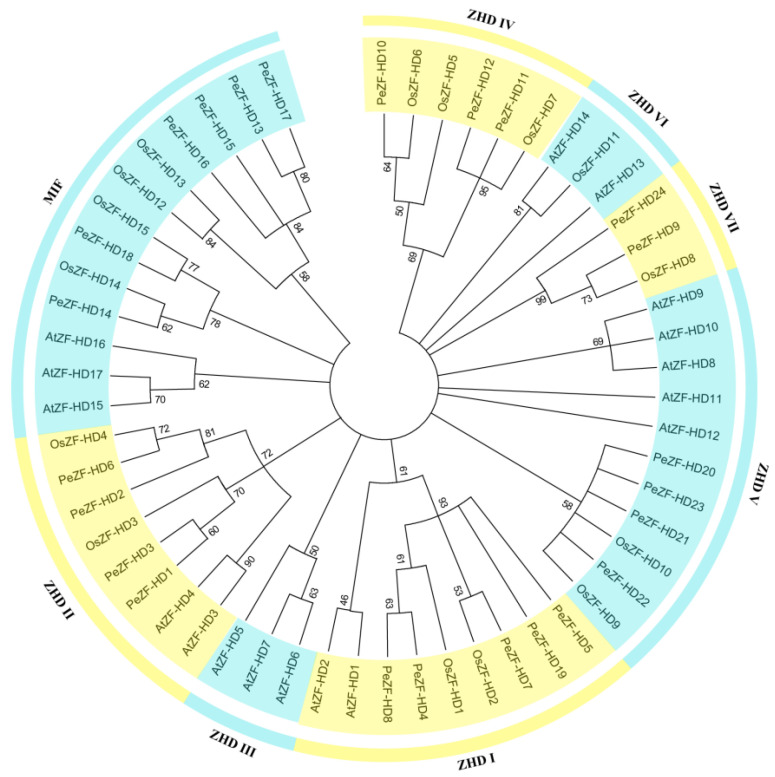
Phylogenetic analysis of ZF-HD proteins from rice, *Arabidopsis* and moso bamboo. The tree was built using the Maximum Likelihood (ML) method with 1000 bootstrap replicates. The branched lines of the subtrees are colored to indicate different subgroups.

To study the evolutionary relationships of PeZF-HDs, a phylogenetic tree consisting of the ZF-HD protein sequences from moso bamboo, rice and *Arabidopsis* was constructed using the Maximum Likelihood (ML) method (Figure 1). The phylogenetic distribution revealed that the PeZF-HD family can be divided into two major groups: MIF and ZHD. ZHD included 43 genes and could be subdivided into seven subgroups: ZHD I, ZHD II, ZHD III, ZHD IV, ZHD V, ZHD VI and ZHD VII. The quantitative distribution of each group of ZF-HD proteins in moso bamboo, rice and *Arabidopsis* were counted (Table S4). Compared to MIF, ZHD had more members in moso bamboo, rice and *Arabidopsis*. The number of ZHD V genes was the highest, with four *PeZF-HD*, two *OsZF-HD* and five *AtZF-HD* genes. Phylogenetic analysis of ZF-HDs in three species showed that PeZF-HDs shared more sequence similarity with OsZF-HDs than with AtZF-HDs (Figure 1).

### 2.3. Conserved Motifs and Gene Structure Analysis of the Moso Bamboo ZF-HD Gene Family

**Figure 2:**
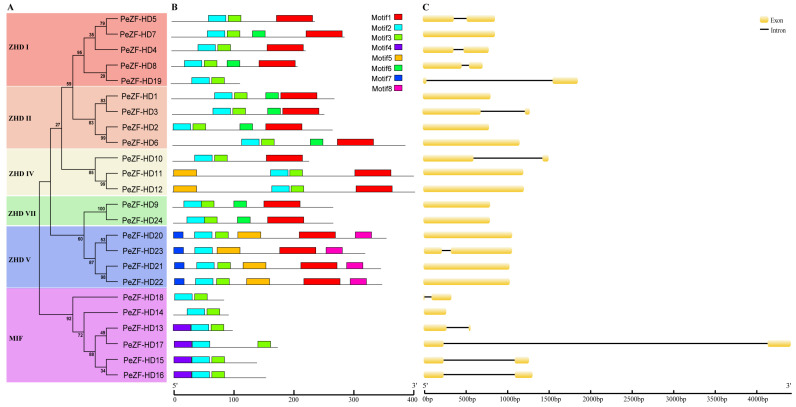
Phylogenetic relationships, gene structures and conserved domains of *PeZF-HD*s. (**A**) ML phylogenetic tree of PeZF-HDs. (**B**) Motif distributions in PeZF-HDs. The colored boxes represent motifs 1–8. Scale bar: 100 amino acids. (**C**) Exon–intron distribution of *PeZF-HD*s. The black lines and yellow boxes represent introns and exons, respectively. Scale bar: 500 bp.

To investigate the structural diversity of the *PeZF-HD* genes, a phylogenetic tree using only the full-length PeZF-HD protein sequences was constructed. Phylogenetic analysis showed that the PeZF-HDs could be categorized into six classes, which was consistent with the phylogenetic tree data among moso bamboo, rice and *Arabidopsis* (Figure 2A). MEME was used to determine differences in the PeZF-HD proteins. Eight conserved motifs were identified and named motifs 1–8 (Figure 2B). Motif 1 was the largest motif and was composed of 60 amino acids. Motif 2 and Motif 3, which were the components of the ZF-HD_dimer domain, were the most common motifs. Motif 2 was present in all PeZF-HDs, whereas Motif 3 was absent in PeZF-HD23. Motif 5 was present in ZHD IV and ZHD V. Motifs 7 and 8 were found exclusively in ZHD V, whereas Motif 4 was detected specifically in MIF. PeZF-HDs within the same class showed similar motif distributions, suggesting that they have functional similarities. Furthermore, the motif distribution in MIF differed greatly from that in the other classes, as it had only two to three motifs (motifs 2, 3 and 4) and the shortest sequence length. These motif distribution differences were probably results of the functional diversity of *PeZF-HD*s.

The exon–intron organizations were analyzed with an online GSDS tool. As shown in Figure 2C, half of the *PeZF-HD*s were intronless and the remaining half had one intron. Most *PeZF-HD*s in the same class had similar intron–exon structures. For example, the *PeZF-HD*s in ZHD V (except for *PeZF-HD23*) all contained no introns. However, the intron–exon structure was not always conserved in most sister gene pairs. For instance, *PeZF-HD5/-7* and *PeZF-HD1/-3* had different numbers of introns and exons. Moreover, all *PeZF-HD*s were less than 2 kb in length, except for *PeZF-HD17* (4.4 kb).

### 2.4. Chromosome Distribution and Gene Duplication of the PeZF-HD Genes

**Figure 3:**
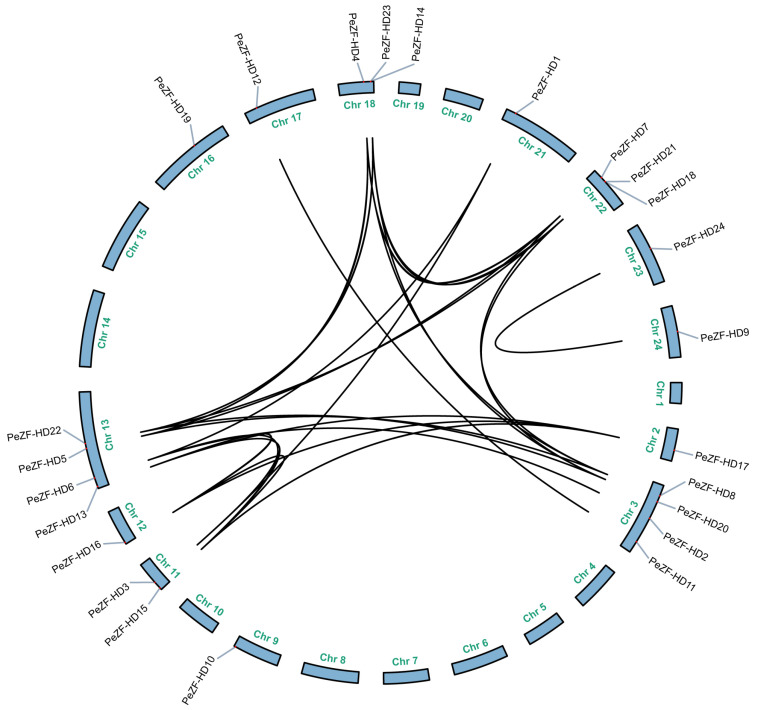
Chromosome distribution and syntenic relationships of *PeZF-HD*s. Chromosomes are shown in a circular form. The duplicate *PeZF-HD* gene pairs are connected by black lines.

The 24 *PeZF-HD*s were unevenly distributed across the 12 chromosome scaffolds based on their location in the moso bamboo genome (Figure 3). Chromosomes 3 and 13 contained the largest number of *PeZF-HD* members (four and four genes, respectively), followed by chromosomes 18 and 22 (three and three genes, respectively). Both chromosomes 11 and 12 contained two genes, while chromosomes 2, 9, 16, 17, 23 and 24 possessed only one gene, and no *ZF-HD*s were present on the twelve remaining moso bamboo chromosomes. Eighteen *PeZF-HD*s were located on the positive-strand chromosomes, while the remaining six genes were positioned on the negative-strand chromosomes. To further investigate the mechanism of the *PeZF-HD* gene family expansion, the potential duplication events of *PeZF-HD*s were identified with the MCScanX program. All *PeZF-HD*s appeared to have arisen from WGD or segmental duplications, except for *PeZF-HD19*, which underwent dispersed duplication (Table S5). No tandem duplication event was found in *PeZF-HD*s.

To estimate the selection constraints of the duplicated *PeZF-HD* genes, the Ka, Ks replacement rate and Ka/Ks ratios of the twenty-four duplication pairs were calculated and listed in Table S6. The Ka/Ks ratios of these paralogous pairs ranged from 0.106 to 0.679, with an average value of 0.375, suggesting that these genes have undergone strong purifying selection during evolution. The Ks values of the *PeZF-HD* gene pairs varied from 0.099 to 0.639, indicating that a large-scale *PeZF-HD* gene duplication event occurred as early as 7.65–49.14 million years ago (MYA). The divergence for most *PeZF-HD* gene pairs (16 of 24) was about 16.51 to 49.14 MYA, which differs from the moso bamboo whole-genome duplication 7–12 MYA, suggesting that the *PeZF-HD* gene underwent other ancient whole-genome duplication events earlier.

### 2.5. Analysis of Cis-Regulatory Elements in PeZF-HD Promoters

**Figure 4:**
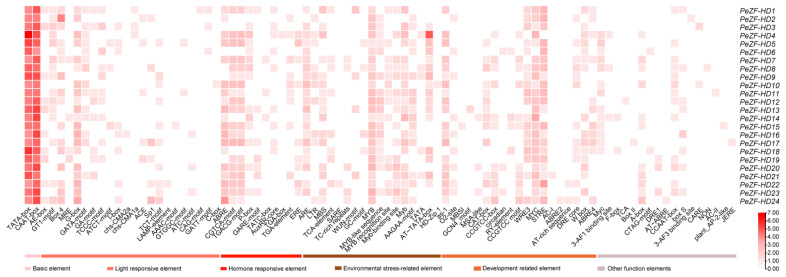
The cis-acting elements of *PeZF-HD*s promoters. The closed boxes of different colors represent different kinds of cis-acting elements.

To explore the potential functions of *PeZF-HD*s, the cis-regulatory elements at the promoter regions were investigated. Besides basic elements, cis-elements responsive to light, development, environmental stress and hormones were found in *PeZF-HD* promoters (Figure 4 and Table S1). A light-responsive element, with the largest proportion in *PeZF-HD* promoters, was represented by as many as 22 types. Each *PeZF-HD* promoter contained between 3 and 10 types. The elements related to development were also analyzed, such as the CAT-box, CCGTCC-box, RY-element, O2-site and GCN4 motif. With regard to environmental stress-related elements, we mainly investigated the elements related to low temperature stress (LTR), drought induction (MBS), salicylic acid response (TCA-element and SARE), and defense and stress response (TC-rich repeats). Interestingly, all *PeZF-HD* promoters contained the MYB element. Numerous elements in the *PeZF-HD* promoters were related to the response to hormones, like ABA (ABRE), MeJA (TGACG-motif and CGTCA-motif), auxin (AuxRR-core, TGA-box and TGA-element) and gibberellic acid (P-box, GAREmotif and TATC-box). Among them, the ABRE was the most abundant element in the *PeZF-HD* promoters, as it was found in 22 promoters. These results indicate that *PeZF-HD*s might be associated with moso bamboo development and have different roles in response to different stresses and hormone signals.

### 2.6. Expression Profiles of PeZF-HD Genes against Different Abiotic Stresses

**Figure 5:**
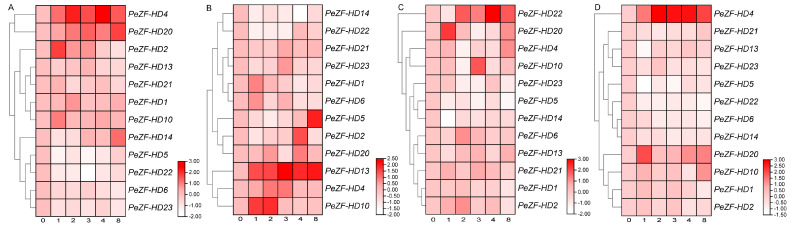
Heatmap and cluster of *PeZF-HD*s expressions under cold (**A**), Nacl (**B**), PEG (**C**) and ABA (**D**) treatment. The relative expression levels of *PeZF-HD*s under these treatments are shown by fold-change values and converted to log2 format compared to the control. The x-axis represents the days of treatment. The color scale at the right of each heatmap represents relative expression levels. The qPCR results were obtained based on three biological and three technical replicates.

The analysis of potential cis-acting elements indicated that *PeZF-HD*s may play roles in plant responses to multiple environmental stresses. Based on the published transcriptome data, twelve *PeZF-HD*s with significantly different expressions were screened. Their expression profiles under cold, salt and drought stresses were determined by qPCR analysis (Figure 5). For moso bamboo seedlings treated with cold stress, the expression of *PeZF-HD2*, *4* and *20* displayed significant up-regulation, while rapid down-regulation was observed for *PeZF-HD5* and *22*. Under salt stress, *PeZF-HD14* was repressed, whereas *PeZF-HD4*, *10* and *13* were induced. In the drought stress, the expression of *PeZF-HD5* and *14* was down-regulated and *PeZF-HD4*, *10*, *20* and *22* were up-regulated. *PeZF-HD4* was significantly up-regulated under cold, salt and drought stresses. The expression of *PeZF-HD14* was reduced and *PeZF-HD10* was induced in both drought and salt stresses, while that of *PeZF-HD5* was down-regulated and *PeZF-HD20* was up-regulated in both cold and drought stresses. Interestingly, some *PeZF-HD*s exhibited inverse gene expression profiles in response to cold, salt and drought treatments. For instance, *PeZF-HD22* was reduced under cold treatment, but was induced under salt and drought treatment. Moreover, the expression of *PeZF-HD22* first decreased and then increased under cold, salt or drought treatments. The plant hormone ABA acts in plant stress signal response and plant growth. Thus, the expression profiles of the twelve *PeZF-HD*s under ABA treatment were also analyzed. Most of the *PeZF-HD*s signals were dramatically up-regulated in response to ABA. Eight of twelve *PeZF-HD*s (*PeZF-HD1*, *2*, 4, *10*, *13*, *20*, *21* and *23*) were up-regulated to varying degrees with this treatment. We concluded the potentially functional *ZF-HD* genes of different species in response to various environmental stresses (Table S7), and the expressions of most *PeZF-HD*s showed similar responses to various environmental stresses in different species. However, the diverse *PeZF-HDs* may differ in their modes of regulation in moso bamboo under abiotic stress.

## 3. Discussion

Plants often face adverse environmental conditions during their lifetime, such as drought, high salinity and cold, which affect physiological and biochemical processes and thus inhibit plant growth and development. To this end, plants have evolved a sophisticated stress resistance system regulated by a series of genes. To date, ZF-HDs have attracted increasing attention in applied and plant basic sciences. ZF-HD family genes are only existent in plants and play crucial roles in plant development and abiotic stress response [3]. The structural characteristics and functions of the ZF-HD gene family have been studied in various plants, such as *Arabidopsis*, rice, maize, tomato and Chinese cabbage [11,12,13,14,18]. However, ZF-HD genes have not been studied in moso bamboo.

Based on the moso bamboo genome, 24 *ZF-HD* genes (18 *ZHD* genes and 6 *MIF* genes) were identified and their physical and chemical properties, conserved motifs, gene evolution and expression were analyzed in this study. The amino acid length of ZF-HDs in moso bamboo ranged from 83 to 403, which was similar to that in rice (105–417). The number of *PeZF-HD*s was more than that of rice and *Arabidopsis*, similar to that of maize and tomato, and less than that of Chinese cabbage [11,12,13,14,18]. The genome size of moso bamboo (2021 Mb) is larger than that of *Arabidopsis* (164 Mb), rice (441 Mb), tomato (950 Mb) and Chinese cabbage (283.8 Mb), and comparable to that of maize (2300 Mb) [25,26,27,28,29]. These results indicate that the number of *ZF-HD*s has no relationship to the plant’s genome size, but may be related to a gene duplication event (Table S5).

*PeZF-HD*s were classified into eight groups based on sequence homology and classification related to rice and *Arabidopsis*, different from the seven subfamilies in alfalfa and cucumber [30,31]. Phylogenetic analysis showed that all PeZF-HD subgroups had at least one homolog of rice ZF-HDs (Figure 1), indicating that the PeZF-HDs shared more sequence similarity with OsZF-HDs, and the subgroups of ZF-HDs were usually conserved in monocotyledonous plants. The groups of *PeZF-HD*s lacked the ZHD III and ZHD VI groups according to classification in relation to rice and *Arabidopsis*. Rice and *Arabidopsis* ZF-HDs were found in ZHD VI, but only *Arabidopsis* ZF-HDs were found in ZHD III, suggesting the protein divergence between monocots and dicots. The number of each group in moso bamboo was different compared to rice and *Arabidopsis*. *ZF-HD*s in plants with different gene numbers in different groups may be under different evolutionary constraints. Furthermore, MIF proteins of moso bamboo, rice and *Arabidopsis* form a phylogenetically distinct clade with ZHD proteins, indicating that the structural divergence between MIF and ZHD genes may be derived from ZHDs after losing the HD domain or may originate from MIFs after gaining the HD domain [4].

Conserved motifs and structural analysis showed that *PeZF-HD*s in the same class were similar in structure (Figure 2), suggesting that PeZF-HD members may be functionally conserved during their evolution. Motifs 2 and 3 were the essential components of the ZF-HD_dimer domain and existed in all 24 *PeZF-HD*s, implying that these two motifs were crucial for *PeZF-HD*s functions. The intron–exon analysis exhibited that half of *PeZF-HD*s lacked introns, and the remaining twelve *PeZF-HD*s had one intron, which reflected intron gain and was almost consistent with the *ZF-HD*s in tomato [13]; yet, most plant *ZF-HD*s are intronless [11,12,14,18]. Precise and complete intron gains and losses stimulate the production of new genes, based on previous reports [32]. The variable intron–exon structures of *ZF-HD*s in moso bamboo compared with that in other plants suggested that there existed structural divergence among *ZF-HD*s in moso bamboo. Moreover, the similar exon–intron organization in different subfamilies suggests that these genes were highly conserved during evolution. Thus, different motifs and exon–intron organizations may provide the structural basis for the diverse functions of PeZF-HDs. These results, combined with phylogenetic analysis, confirmed the reliability of group classification and the similar biological functions of *PeZF-HD*s within the same group.

Gene duplication patterns may reveal the generational types of genes and evolutionary ways of gene functions [33]. Almost all *PeZF-HD*s (except for *PeZF-HD19*) were duplicated through WGD or segmental duplication events, suggesting that WGD or segmental duplication played a key role in the expansion of *PeZF-HD*s, which was similar to the *ZF-HD*s in other plants, like tomato and Chinese cabbage [13,18]. The Ka/Ks values of paralogous genes were high at 0.106-0.679 (the average value was 0.375), implying purifying selection and a powerful selection constraint in *PeZF-HD*s (Table S6). Intra-genomic collinearity analysis of *PeZF-HD*s was performed and 24 pairs of duplicated genes were found in the moso bamboo genome (Figure 3). Most duplication pairs had conserved motifs and gene structures, and only a few had a certain degree of differentiation (Figure 2). For example, *PeZF-HD*1 was intronless, while its corresponding *PeZF-HD3* had one intron, which could be due to the gaining of a single intron in the gene structure during evolution. Most divergence times of the *PeZF-HD* gene pairs occurred from 16.51 to 49.14 MYA, which differs from the recent whole-genome duplication time of moso bamboo (7-12 MYA) [20], suggesting that the effects of other earlier ancient whole-genome duplication events also influenced the expansion of *PeZF-HD* genes.

The different *cis*-elements on promoters likely led to functional differentiation in *PeZF-HD*s. Elements related to stress responses were found in *PeZF-HD* promoters, including MYB, MBS, LTR, TCA-element, TC-rich repeats and ABRE (Table S1), which are directly related to ABA, drought, cold and salt stress responses. *ZF-HD* genes were reported to be involved in various abiotic stresses and ABA treatment, such as salt and drought stress [12,17,19,34]. Moreover, *ZF-HD* genes in alfalfa also showed similarly decreased expression levels under alkaline stress [31]. Previous studies investigated the expression profiles of *ZF-HD* genes under multiple stress conditions with transcriptome analysis and qPCR [11,19,31]. In this study, the qPCR results showed that *PeZF-HD*s 5, 4 and 6 were differentially expressed under cold, salt and PEG stresses, respectively, which also verified the results of the *cis*-elements analysis. Most differentially expressed *PeZF-HD*s were also significantly changed under ABA treatment (Figure 5). Particularly, overlapping responses of *PeZF-HD*s to multiple stresses were found, like *PeZF-HD10*, which was induced under drought and salt stresses, helping the potential hub genes in moso bamboo to acclimate to stressful conditions. The overexpression of *AtZHD1* improved drought tolerance in *Arabidopsis* [15] and the overexpression of *CqZF-HD14* enhanced the defense system of quinoa under drought [21]. *PeZF-HD4*, the homologous gene to *AtZF-HD1*, was up-regulated under cold, salt, PEG and ABA treatments. *PeZF-HD5*, another homologous gene to *AtZF-HD1*, was up-regulated under salt treatment. *AtZHD4* was induced under cold, salt and drought treatments [35]. The expression of *AtZHD4* homolog *PeZF-HD2* also increased under cold, salt and drought treatments. These results showed that *PeZF-HD*s may play important roles in response to various abiotic stresses, most of which may be mediated by ABA signaling. The above results provide an information base for selecting candidate genes and promote further functional investigations of biotic stress resistance in moso bamboo.

## 4. Materials and Methods

### 4.1. Plant Material and Treatments

The moso bamboo (*Phyllostachys edulis*) was selected for this experiment as it has completed whole-genome sequencing. The germinated seeds were planted in pots with medium (soil and vermiculite, 1:1) in a growth room under a controlled environment (16 h light at 23 °C/8 h dark at 18 °C). Two-month-old seedlings were treated with 20% PEG6000, 100 µM ABA and 250 mM NaCl, respectively. For the low temperature treatment, the seedlings were transferred to 4 °C conditions. The leaf samples were collected along a continuous time course (0, 1, 2, 3, 4, 8 h) with three biological replicates and then stored at −80 °C. Total RNA was extracted by the RNAsimple Total RNA Kit (Tiangen, Beijing, China) and residual genomic DNA was removed with DNase I (Qiagen, Valencia, CA, USA) following the manufacturer’s instructions.

### 4.2. Identification of ZF-HD Genes in Moso Bamboo

The *P. edulis* genome sequence was downloaded from the Bamboo Genome Database (http://202.127.18.221/bamboo/index.php, accessed on 8 April 2023). The ZF-HD gene sequences of *Oryza* and *Arabidopsis* were obtained from the rice genome annotation project and the *Arabidopsis* information resource based on published studies, respectively [4,11]. Members of the *PeZF-HD* genes were identified by BLASTP searches and Hidden Markov Model (HMM) searches. Protein sequences of *Oryza* and *Arabidopsis ZF-HD*s were used as queries to make BLASTP searches (*E* value < 1 × 10^−10^). The annotation file of ZF-HD_dimer domain (PF04770) was applied to build the HMM profile against the *P. edulis* protein dataset (*E* value < 1 × 10^−10^). The ZF-HD proteins were further identified by InterProScan (https://www.ebi.ac.uk/interpro/, accessed on 8 April 2023). A total of 24 *PeZF-HD* genes were identified in the moso bamboo genome and redundant sequences were removed.

### 4.3. Sequence Analysis

The molecular weight, length and isoelectric point of PeZF-HD proteins were analyzed by the ExPASy website (https://web.expasy.org, accessed on 20 April 2023) (Table 1). The CDS and genomic sequences of *PeZF-HD* were aligned to analyze the exon–intron structures using the GSDS online program [36]. The protein sequences were analyzed by the MEME program (http:/meme.nbcr.net/meme/intro.html, accessed on 20 April 2023) with the following parameters: the maximum number of motifs was 8, and the width of the optimum motif was 6–300. The subcellular location of the putative PeZF-HD proteins was predicted by Plant-mPLoc (http://www.csbio.sjtu.edu.cn/bioinf/plant-multi/, accessed on 20 April 2023).

### 4.4. Phylogenetic Analysis and Classification

The ZF-HD protein sequences of rice and *Arabidopsis* were obtained from the TIGR-Rice Genome Annotation Project and *Arabidopsis* Information resource, respectively [37,38]. The full-length amino acid sequences were aligned using Clustal X according to our previous report [39]. The phylogenetic tree was built using MEGA 7.0 software with the Maximum Likelihood (ML) algorithm and 1000 bootstrap replicates.

### 4.5. Analysis of Cis-Element in the Promoter Regions

To predict *cis*-regulatory elements in promoters of *PeZF-HD* genes, 2000 bp upstream sequences of the translational start codon were detected by PlantCARE (http://bioinformatics.psb.ugent.be/webtools/plantcare/html/, accessed on 10 May 2023). The heat map was made using TBtools by counting the *cis*-regulatory elements on each promoter (Table S1) [40].

### 4.6. Chromosome Localization and Gene Duplication

The structural and positional information of *PeZF-HD*s on the chromosomes of moso bamboo was downloaded from the moso bamboo genome database [24]. The positions of *PeZF-HD*s on the moso bamboo chromosomes were drawn using the MapGene2Chromosome2 web tool (http://mg2c.iask.in/, accessed on 19 May 2023) [41]. The syntenic relationships of the *ZF-HD*s in moso bamboo, rice and *Arabidopsis* were analyzed using MCScanX with default parameters [42]. The results of the chromosomal location and synteny relationships were visualized by Circos software (http://mkweb.bcgsc.ca/circos) [43]. The duplication events of the *PeZF-HD*s were analyzed using MCScanX following the default parameters.

### 4.7. Calculation of Ka/Ks Ratios

The non-synonymous (Ka) and synonymous (Ks) replacement rates and Ka/Ks values of the duplicated *ZF-HD* gene pairs were calculated using KaKs_Calculator software v2.0 [44]. Evolutionary divergence times of the moso bamboo *ZF-HD* gene family were calculated by the bamboo-specific divergence time formula (T = Ks/2λ, λ = 6.5 × 10^−9^). The selection pressure of the duplicate gene pairs was determined by the Ka/Ks ratio [45].

### 4.8. Quantitative Real-Time PCR Expression Analysis

Total RNA extraction, cDNA synthesis and qPCR reaction were performed according to our protocol [39]. To confirm the specificity of the qPCR reactions, a melting curve was implemented. The *PeNTB* gene was used as an internal standard [46]. Data were calculated by the 2^−ΔΔCT^ method [47]. The heat map was made by TBtools with a normalized row scale [40]. The expression results were analyzed by three independent biological replicates. Primers are shown in Table S2. The published transcriptome data of moso bamboo under PEG, NaCl and ABA treatments were obtained from the Gene Expression Omnibus database with the accession number GSE169067 (http://www.ncbi.nlm.nih.gov/geo, accessed on 10 April 2023).

## 5. Conclusions

In this study, genome-wide detection and analyses of the *PeZF-HD* gene family were conducted in moso bamboo. The twenty-four PeZF-HDs were classified into two main groups and eight subgroups based on the grouping principles of the rice and *Arabidopsis* ZF-HD family, which was further supported by their similar motif compositions and exon–intron structures. WGD or segmental duplications were indicated to have been the major driving force for the expansion of *PeZF-HD*s. Multiple *PeZF-HD* expressions were stimulated by various types of abiotic stress. Our results provide a valuable basis for the functional study of ZF-HD genes and facilitate the identification of candidate genes in moso bamboo stress response networks, which may shed a light on moso bamboo cultivation.

## Figures and Tables

**Table 1:** Detailed information of ZF-HDs identified in moso bamboo genome.

Gene Name	CDS (bp)	Size (aa)	Mass (KDa)	pI	Domain	Exon Number	Location	Predicted Localization
*PeZF-HD1*	816	271	29.36	9.41	ZF-HD	1	chromosomes 12:13827712:13828527:+	Nucleus
*PeZF-HD2*	801	266	28.61	9.05	ZF-HD	1	chromosomes 3:49715743:49719049:+	Nucleus
*PeZF-HD3*	762	253	27.04	8.50	ZF-HD	2	chromosomes 11:13377647:13378939:+	Nucleus
*PeZF-HD4*	672	223	23.46	8.78	ZF-HD	2	chromosomes 18:35699501:35700292:+	Nucleus
*PeZF-HD5*	717	238	24.80	6.36	ZF-HD	2	chromosomes 13:57959543:57960412:+	Nucleus
*PeZF-HD6*	1164	387	40.65	8.59	ZF-HD	1	chromosomes 13:15365059:15366222:-	Nucleus
*PeZF-HD7*	867	288	29.90	6.75	ZF-HD	1	chromosomes 22:13492044:13492910:+	Nucleus
*PeZF-HD8*	633	210	22.79	8.41	ZF-HD	2	chromosomes 3:15339955:15340674:+	Nucleus
*PeZF-HD9*	804	267	27.82	8.49	ZF-HD	1	chromosomes 24:36670935:36671738:-	Nucleus
*PeZF-HD10*	684	227	24.51	8.99	ZF-HD	2	chromosomes 9:63441848:63443360:+	Nucleus
*PeZF-HD11*	1206	401	43.36	6.58	ZF-HD	1	chromosomes 3:87050975:87052180:-	Nucleus
*PeZF-HD12*	1212	403	43.21	6.35	ZF-HD	1	chromosomes 17:17852519:17853730:+	Nucleus
*PeZF-HD13*	300	99	10.57	8.60	ZF-HD	2	chromosomes 13:2989657:2990222:-	Nucleus
*PeZF-HD14*	279	92	10.17	6.79	ZF-HD	1	chromosomes 18:45423575:45424757:-	Nucleus
*PeZF-HD15*	417	138	15.17	10.30	ZF-HD	2	chromosomes 11:2971936:2974744:+	Nucleus
*PeZF-HD16*	462	153	16.81	9.06	ZF-HD	2	chromosomes 12:2567371:2568681:+	Nucleus
*PeZF-HD17*	519	172	18.32	8.95	ZF-HD	2	chromosomes 2:29976915:29981334:+	Nucleus
*PeZF-HD18*	252	83	8.59	6.86	ZF-HD	2	chromosomes 22:21025823:21026158:-	Nucleus
*PeZF-HD19*	348	115	11.41	5.90	ZF-HD	2	chromosomes 16:73535364:73537228:+	Nucleus
*PeZF-HD20*	1065	354	36.91	7.26	ZF-HD	1	chromosomes 3:24470226:24471290:+	Nucleus
*PeZF-HD21*	1035	344	35.64	7.71	ZF-HD	1	chromosomes 22:20913172:20914206:+	Nucleus
*PeZF-HD22*	1041	346	35.73	6.97	ZF-HD	1	chromosomes 13:64992849:64993889:+	Nucleus
*PeZF-HD23*	957	318	33.41	6.73	ZF-HD	2	chromosomes 18:45322937:45323998:+	Nucleus
*PeZF-HD24*	801	266	27.63	8.14	ZF-HD	1	chromosomes 23:37483279:37484079:+	Nucleus

## Data Availability

The data is contained within the manuscript and Supplementary Materials.

## Supplementary Materials

The following supporting information can be downloaded at: https://www.mdpi.com/article/10.3390/plants12234064/s1, Table S1: Detailed information of *ZF-HDs* identified in moso bamboo genome; Table S2: Catergory information of cis-elements in *PeZF-HDs* promoters; Table S3: Primers used in the paper; Table S4: Identification of conserved domains in PeZF-HD proteins using the SMART tool; Table S5: Quantitative distribution of each group of *ZF-HDs* in moso bamboo, rice and *Arabidopsis*; Table S6: The mode of gene duplication in *PeZF-HDs*; Table S7: The KaKs ratios and estimated divergence times for duplicate gene pairs of *PeZF-HDs*. References [48,49] are cited in the supplementary materials.

## References

[1] Pereira A. (2016). Plant abiotic stress challenges from the changing environment. Front. Plant Sci..

[2] Amorim L.L.B., da Fonseca Dos Santos R., Neto J.P.B., Guida-Santos M., Crovella S., Benko-Iseppon A.M. (2017). Transcription factors involved in plant resistance to pathogens. Curr. Protein Pept. Sci..

[3] Tan Q.K., Irish V.F. (2006). The Arabidopsis zinc finger-homeodomain genes encode proteins with unique biochemical properties that are coordinately expressed during floral development. Plant Physiol..

[4] Hu W., dePamphilis C.W., Ma H. (2008). Phylogenetic analysis of the plant-specific zinc finger-homeobox and mini zinc finger gene families. J. Integr. Plant Biol..

[5] Windhövel A., Hein I., Dabrowa R., Stockhaus J. (2001). Characterization of a novel class of plant homeodomain proteins that bind to the C4 phosphoenolpyruvate carboxylase gene of Flaveria trinervia. Plant Mol. Biol..

[6] Mukherjee K., Brocchieri L., Bürglin T.R. (2009). A comprehensive classification and evolutionary analysis of plant homeobox genes. Mol. Biol. Evol..

[7] Ariel F.D., Manavella P.A., Dezar C.A., Chan R.L. (2007). The true story of the HD-Zip family. Trends Plant Sci..

[8] Klug A., Schwabe J.W. (1995). Protein motifs 5. Zinc fingers. FASEB J..

[9] Krishna S.S., Majumdar I., Grishin N.V. (2003). Structural classification of zinc fingers: Survey and summary. Nucleic Acids Res..

[10] Hu W., Ma H. (2006). Characterization of a novel putative zinc finger gene MIF1: Involvement in multiple hormonal regulation of Arabidopsis development. Plant J..

[11] Jain M., Tyagi A.K., Khurana J.P. (2008). Genome-wide identification, classification, evolutionary expansion and expression analyses of homeobox genes in rice. FEBS J..

[12] Figueiredo D.D., Barros P.M., Cordeiro A.M., Serra T.S., Lourenço T., Chander S., Oliveira M.M., Saibo N.J. (2012). Seven zinc-finger transcription factors are novel regulators of the stress responsive gene *OsDREB1B*. J. Exp. Bot..

[13] Khatun K., Nath U.K., Robin A.H.K., Park J.I., Lee D.J., Kim M.B., Kim C.K., Lim K.B., Nou I.S., Chung M.Y. (2017). Genome-wide analysis and expression profiling of zinc finger homeodomain (ZHD) family genes reveal likely roles in organ development and stress responses in tomato. BMC Genom..

[14] Islam M.A.U., Nupur J.A., Khalid M.H.B., Din A.M.U., Shafiq M., Alshegaihi R.M., Ali Q., Kamran Z., Manzoor M., Haider M.S. (2022). Genome-wide identification and in silico analysis of ZF-HD transcription factor genes in *Zea mays* L.. Genes.

[15] Tran L.S., Nakashima K., Sakuma Y., Osakabe Y., Qin F., Simpson S.D., Maruyama K., Fujita Y., Shinozaki K., Yamaguchi-Shinozaki K. (2007). Co-expression of the stress-inducible zinc finger homeodomain ZFHD1 and NAC transcription factors enhances expression of the *ERD1* gene in *Arabidopsis*. Plant J..

[16] Niu H., Xia P., Hu Y., Zhan C., Li Y., Gong S., Li Y., Ma D. (2021). Genome-wide identification of ZF-HD gene family in Triticum aestivum: Molecular evolution mechanism and function analysis. PLoS ONE.

[17] Zhao T.T., Wang Z.Y., Bao Y.F., Zhang X.C., Yang H.H., Dong Z.Y., Jiang J.B., Zhang H., Li J.F., Chen Q.S. (2019). Downregulation of SL-ZH13 transcription factor gene expression decreases drought tolerance of tomato. J. Integr. Agric..

[18] Wang W., Wu P., Li Y., Hou X. (2016). Genome-wide analysis and expression patterns of ZF-HD transcription factors under different developmental tissues and abiotic stresses in Chinese cabbage. Mol. Genet. Genom..

[19] Xing L., Peng K., Xue S., Yuan W., Zhu B., Zhao P., Wu H., Cheng Y., Fang M., Liu Z. (2022). Genome-wide analysis of zinc finger-homeodomain (ZF-HD) transcription factors in diploid and tetraploid cotton. Funct. Integr. Genom..

[20] Liu M., Wang X., Sun W., Ma Z., Zheng T., Huang L., Wu Q., Tang Z., Bu T., Li C. (2019). Genome-wide investigation of the *ZF-HD* gene family in Tartary buckwheat (*Fagopyrum tataricum*). BMC Plant Biol..

[21] Sun W., Wei J., Wu G., Xu H., Chen Y., Yao M., Zhan J., Yan J., Wu N., Chen H. (2022). CqZF-HD14 enhances drought tolerance in quinoa seedlings through interaction with CqHIPP34 and CqNAC79. Plant Sci..

[22] Peng Z., Lu Y., Li L., Zhao Q., Feng Q., Gao Z., Lu H., Hu T., Yao N., Liu K. (2013). The draft genome of the fast-growing non-timber forest species moso bamboo (*Phyllostachys heterocycla*). Nat. Genet..

[23] Wei Q., Guo L., Jiao C., Fei Z., Chen M., Cao J., Ding Y., Yuan Q. (2019). Characterization of the developmental dynamics of the elongation of a bamboo internode during the fast growth stage. Tree Physiol..

[24] Zhao H., Gao Z., Wang L., Wang J., Wang S., Fei B., Chen C., Shi C., Liu X., Zhang H. (2018). Chromosome-level reference genome and alternative splicing atlas of moso bamboo (*Phyllostachys edulis*). GigaScience.

[25] Duvick J., Fu A., Muppirala U., Sabharwal M., Wilkerson M.D., Lawrence C.J., Lushbough C., Brendel V. (2008). PlantGDB: A resource for comparative plant genomics. Nucleic Acids Res..

[26] Burr B. (2002). Mapping and sequencing the rice genome. Plant Cell.

[27] Filichkin S.A., Priest H.D., Givan S.A., Shen R., Bryant D.W., Fox S.E., Wong W.K., Mockler T.C. (2010). Genome-wide mapping of alternative splicing in Arabidopsis thaliana. Genome Res..

[28] Van der Hoeven R., Ronning C., Giovannoni J., Martin G., Tanksley S. (2002). Deductions about the number, organization, and evolution of genes in the tomato genome based on analysis of a large expressed sequence tag collection and selective genomic sequencing. Plant Cell.

[29] Wang X., Wang H., Wang J., Sun R., Wu J., Liu S., Bai Y., Mun J.H., Bancroft I., Cheng F. (2011). The genome of the mesopolyploid crop species *Brassica rapa*. Nat. Genet..

[30] Lai W., Zhu C., Hu Z., Liu S., Wu H., Zhou Y. (2021). Identification and transcriptional analysis of zinc finger-homeodomain (ZF-HD) family genes in cucumber. Biochem. Genet..

[31] He K., Li C., Zhang Z., Zhan L., Cong C., Zhang D., Cai H. (2022). Genome-wide investigation of the *ZF-HD* gene family in two varieties of alfalfa (*Medicago sativa* L.) and its expression pattern under alkaline stress. BMC Genom..

[32] Roy S.W., Penny D. (2007). Patterns of intron loss and gain in plants: Intron loss-dominated evolution and genome-wide comparison of *O. sativa* and *A. thaliana*. Mol. Biol. Evol..

[33] Wang T., Hu J., Ma X., Li C., Yang Q., Feng S., Li M., Li N., Song X. (2020). Identification, evolution and expression analyses of whole genome-wide TLP gene family in *Brassica napus*. BMC Genom..

[34] Shi B., Haq I., Fiaz S., Alharthi B., Xu M., Wang J., Hou W., Feng X. (2022). Genome-wide identification and expression analysis of the ZF-HD gene family in pea (*Pisum sativum* L.). Front. Genet..

[35] Barth O., Vogt S., Uhlemann R., Zschiesche W., Humbeck K. (2009). Stress induced and nuclear localized HIPP26 from Arabidopsis thaliana interacts via its heavy metal associated domain with the drought stress related zinc finger transcription factor ATHB29. Plant Mol. Biol..

[36] Hu B., Jin J., Guo A.Y., Zhang H., Luo J., Gao G. (2015). GSDS 2.0: An upgraded gene feature visualization server. Bioinformatics.

[37] Yuan Q., Ouyang S., Liu J., Suh B., Cheung F., Sultana R., Lee D., Quackenbush J., Buell C.R. (2003). The TIGR rice genome annotation resource: Annotating the rice genome and creating resources for plant biologists. Nucleic Acids Res..

[38] Swarbreck D., Wilks C., Lamesch P., Berardini T.Z., Garcia-Hernandez M., Foerster H., Li D., Meyer T., Muller R., Ploetz L. (2008). The Arabidopsis Information Resource (TAIR): Gene structure and function annotation. Nucleic Acids Res..

[39] Huang F., Liu T., Hou X. (2018). Isolation and functional characterization of a floral repressor, BcMAF1, from Pak-choi (*Brassica rapa* ssp. chinensis). Front. Plant Sci..

[40] Chen C., Chen H., Zhang Y., Thomas H.R., Frank M.H., He Y., Xia R. (2020). TBtools: An integrative toolkit developed for interactive analyses of big biological data. Mol. Plant.

[41] Jiangtao C., Yingzhen K., Qian W., Yuhe S., Daping G., Jing L., Guanshan L. (2015). MapGene2Chrom, a tool to draw gene physical map based on Perl and SVG languages. Yi chuan.

[42] Wang Y., Tang H., Debarry J.D., Tan X., Li J., Wang X., Lee T.H., Jin H., Marler B., Guo H. (2012). MCScanX: A toolkit for detection and evolutionary analysis of gene synteny and collinearity. Nucleic Acids Res..

[43] Krzywinski M., Schein J., Birol I., Connors J., Gascoyne R., Horsman D., Jones S.J., Marra M.A. (2009). Circos: An information aesthetic for comparative genomics. Genome Res..

[44] Zhang Z., Li J., Zhao X.Q., Wang J., Wong G.K., Yu J. (2006). KaKs_Calculator: Calculating Ka and Ks through model selection and model averaging. Proteom. Bioinf..

[45] Lynch M., Conery J.S. (2000). The evolutionary fate and consequences of duplicate genes. Science.

[46] Fan C., Ma J., Guo Q., Li X., Wang H., Lu M. (2013). Selection of reference genes for quantitative real-time PCR in bamboo (*Phyllostachys edulis*). PLoS ONE.

[47] Livak K.J., Schmittgen T.D. (2001). Analysis of relative gene expression data using real-time quantitative PCR and the 2^−ΔΔCT^ Method. Methods.

[48] Bai Y., Kissoudis C., Yan Z., Visser R.G., van der Linden G. (2018). Plant behaviour under combined stress: Tomato responses to combined salinity and pathogen stress. Plant J..

[49] Luo H., Song F., Zheng Z. (2005). Overexpression in transgenic tobacco reveals different roles for the rice homeodomain gene OsBIHD1 in biotic and abiotic stress responses. J. Exp. Bot..

